# Pyoderma Gangrenosum: A Rare Cause of Cutaneous Ulceration and One Easily Misdiagnosed

**DOI:** 10.1155/2016/5971706

**Published:** 2016-09-21

**Authors:** Wedad Abdelrahman, Maureen Y. Walsh, Susannah E. Hoey, Donal O'Kane

**Affiliations:** ^1^Department of Dermatology, Royal Victoria Hospital, Belfast BT12 6BA, UK; ^2^Department of Pathology, Royal Victoria Hospital, Belfast BT12 6BA, UK

## Abstract

Pyoderma gangrenosum (PG) is a rare inflammatory neutrophilic dermatosis often misdiagnosed. It is uncommon in infants and children accounting for 4% of cases. A one-year-old male in paediatric ICU ventilated for bronchopneumonia was referred with ulcerated areas on his neck and axilla corresponding to sites of recent removal of central and arterial lines. Examination revealed areas of deep ulceration with violaceous undermined borders in keeping with PG. This was supported by a skin biopsy showing a neutrophilic infiltrate in the deeper dermis. Topical clobetasol propionate was commenced and a dramatic improvement within 24 hours noted. Blood results showed a leucocytosis of 29.7; a differential WCC showed toxic granulation in neutrophils with myeloid left shift; immunoglobulins showed elevated IgG 23 and IgA 4.86. The elevated WCC made us consider a leukaemic trigger; however, they settled with treatment of the underlying infection. PG in children is more likely to have an atypical distribution involving the head and neck (26.6%) or buttocks (15%). An interesting feature in this case is the presence of pathergy, a term used to describe the induction or exacerbation of PG at sites of iatrogenic or incidental trauma. It is seen in 31% of patients with PG.

## 1. Introduction

Pyoderma gangrenosum (PG) is a rare, inflammatory neutrophilic dermatosis which has been reported in all age groups. It can be easily misdiagnosed thus causing a delay in instigating treatment.

## 2. Case Presentation

**Figure 1:**
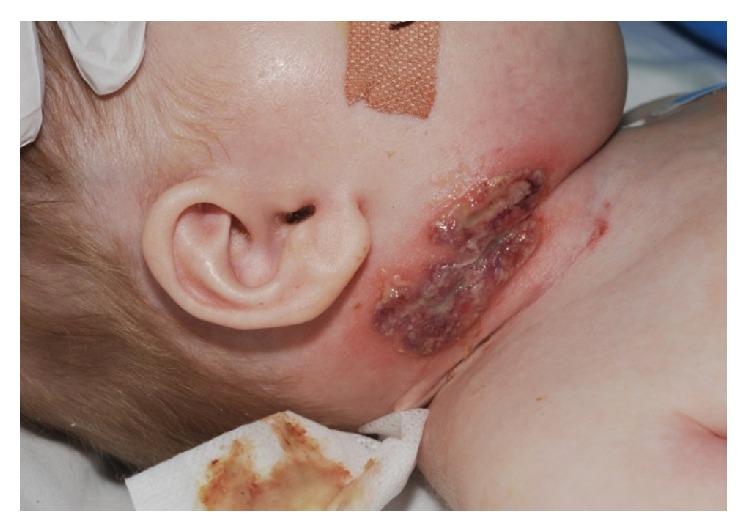
24/12/14: deep violaceous ulcerated area with an undermined border on the right side of neck corresponding to site of arterial line insertion.

**Figure 2:**
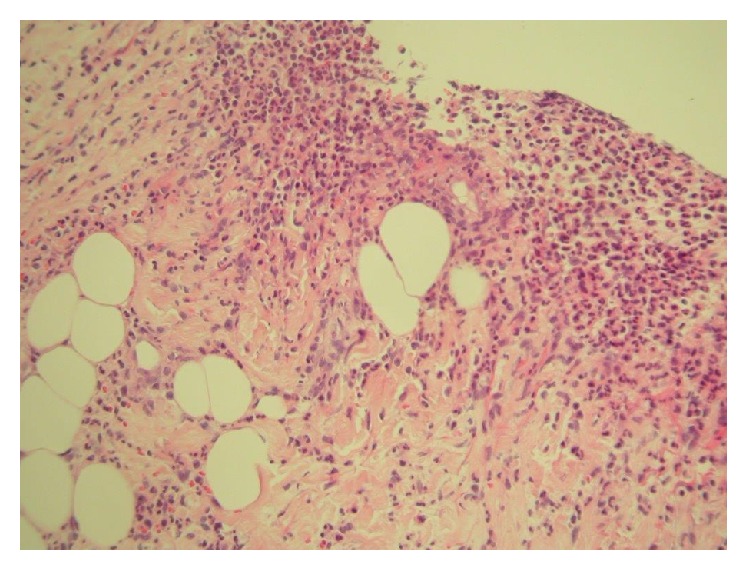
A dense neutrophilic infiltrate in the upper dermis around the dermal blood vessels, in the deeper dermis and the subcutaneous fat (haematoxylin and eosin; original magnification ×25).

**Figure 3:**
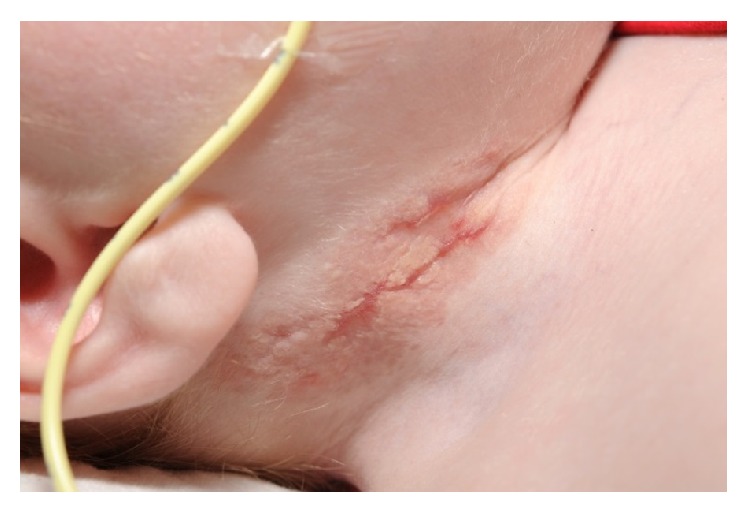
29/12/14: healing with cribriform scarring following treatment with topical clobetasol propionate.

A one-year-old male was referred to Dermatology with nonhealing ulcerated areas on the right side of his neck and axilla corresponding to sites of recent removal of central and arterial lines, respectively. The working diagnosis from the referring doctors was contact allergic eczema to the dressing used to secure the lines. At the time of referral, he was intubated and ventilated in paediatric intensive care receiving intravenous (IV) antibiotic therapy for pneumonia. Sputum was positive for metapneumovirus. He had a significant past medical history including a bicuspid aortic valve, visual impairment, and chromosomal duplication disorder with significant developmental delay. On examination he had deep ulcerated areas with a violaceous undermined border on the right side of the neck (Figure 1) and right axilla in keeping with PG. A skin biopsy (Figure 2) supported this demonstrating a neutrophilic infiltrate in the upper dermis around the dermal blood vessels, in the deeper dermis and the subcutaneous fat. A small number of mononuclear cells were present. There was no evidence of vasculitis. Blood results revealed a marked leucocytosis (41.4 × 10^9^/L); predominantly neutrophils (32.4 × 10^9^/L); elevated C reactive protein 145 mg/L; anaemia; and thrombocytopenia. Blood film examination revealed a myeloid left shift and toxic granulation in neutrophils. Neutrophil integrin expression was normal. IgG and IgA were both elevated at 23 and 4.86 g/L, respectively. Specialist input from Immunology and Haematology was sought and it was felt that both the hyperglobulinaemia and blood film findings were secondary to inflammation and sepsis. Topical therapy with clobetasol propionate was commenced with a dramatic improvement within 24 hours (Figure 3). Skin swabs were taken from line insertion sites and grew* Candida albicans*; this was felt to be of clinical insignificance as the lesions showed a dramatic clinical improvement within 24 hours of applying topical clobetasol propionate. No antifungal therapy was required and subsequent swabs were negative. Interestingly, similar lesions were noted a few days later upon resiting the central line to the left side of his neck. These cleared rapidly with the same treatment. One month later he was readmitted with pneumonia requiring a further course of IV antibiotics but fortunately did not develop any PG lesions. The clinical findings supported by histological features and the dramatic response to potent topical corticosteroid therapy favour a diagnosis of PG induced by pathergy. Furthermore, these lesions healed by forming cribriform scarring which is typical of PG.

## 3. Discussion

The term* pyoderma gangrenosum* is coined from the characteristic appearance of these lesions. They begin as pustules or vesiculopustules that progress to an ulcer with undermined borders which evolves into an enlarging necrotic suppurative ulcer after gangrenous changes in the overlying skin. The lesions last from a few months to years and typically heal with cribriform scarring. PG lesions can be classified into ulcerative, pustular, bullous, vegetative, periosteal, genital, infantile, and extra cutaneous [1].

PG is rare in infants and children with an estimated incidence of about 4% [2]. In 1994, Graham et al. reported the distribution amongst the paediatric population as follows: lower extremities (80%), head (26.1%), buttocks (15%); and in children less than 2 years, involvement of the perianal or genital regions were commonly noted [3]. Although 25–50% of PG cases are idiopathic, an association with underlying systemic disease is recognised and it is paramount to exclude these in patients who are otherwise systemically well. In children the most common association has been with inflammatory bowel disease (IBD): ulcerative colitis > Crohn's disease [2]. Others reported are haematological malignancies such as leukaemia, systemic lupus erythematosus, immunodeficiency disorders, and HIV infection [4]. In a process termed* pathergy*, PG lesions develop at sites of trivial trauma as demonstrated by this case.

The clinical, histological, and laboratory findings in PG are fairly nonspecific, and the diagnosis is usually made once other diagnostic possibilities have been excluded. There are no validated diagnostic criteria for PG; however, the following have been used by some authors [5]:

Major criteria are (must have both)

- rapid progression of a painful ulcer with a violaceous, undermined border,
- other causes of cutaneous ulceration being excluded.

Minor criteria are (must have two)

- history suggestive of pathergy or clinical findings in keeping with cribriform scarring,
- systemic disease associated with PG,
- histology findings: neutrophilic dermal infiltrate/mixed inflammation/lymphocytic vasculitis,
- rapid response to systemic corticosteroid therapy.

The patient in the reported case fulfilled both major criteria and all four minor criteria, but, of note, he responded to potent topical corticosteroid therapy alone without the need for systemic treatment.

The differential diagnosis for PG is wide and includes infection, vasculitis, malignancy, Sweet's syndrome, and dermatitis artefacta. Skin swabs from lesional skin did confirm the presence of* Candida albicans*; however, this was felt to be of clinical insignificance as the affected areas resolved without the need for antifungal therapy. Furthermore, the skin biopsy was not suggestive of an atypical cutaneous infection and excluded a vasculitic process as well as malignancy, the latter being rare in children. Sweet's syndrome, like PG, is a neutrophilic dermatosis, but is characterized by an abrupt onset of a typical skin rash in the form of oedematous erythematous papules and plaques which may become bullous favouring the upper back and posterior neck areas supported by histological features of a neutrophilic infiltrate. These are the two major criteria required for diagnosis. Although this patient had a neutrophilic infiltrate, the rash was clinically not in keeping with Sweet's syndrome. Dermatitis artefacta is also an important differential to consider as PG lesions can have an unusual configuration and when suspected patients should be admitted for close observation and management [6]. Early recognition of PG is paramount as it can prevent unnecessary surgical procedures which may in fact exacerbate the condition further as a result of pathergy [7].

Wound care forms an important aspect in the management of PG and aims to create an optimal environment for wound healing. Topical corticosteroids are commonly the first-line therapy of choice; however, there is a paucity of published literature to support their use. In one study, 5 of 13 patients with limited PG achieved remission with clobetasol propionate [8]. Intralesional (IL) steroids have been shown to be effective but, in patients who demonstrate pathergy, they are best avoided. Patients with extensive lesions or those who fail topical therapy alone will need systemic therapy and oral steroids are generally used first line as they induce a rapid response [9]. A variety of systemic steroid sparing agents have been implicated to be beneficial in the treatment of PG including Ciclosporin, Azathioprine, Dapsone, oral tetracyclines, and antitumour necrosis factor- (TNF-) *α* agents such as Infliximab, though evidence is restricted to case reports and case series with clinical trials and comparative studies lacking. Two cases demonstrating success with Infliximab have been reported in the literature. The first of these, by Rajan et al. [10], were failed oral Prednisolone; IV Methylprednisolone; Ciclosporin; and Dapsone. The other case required the addition of Ciclosporin to Infliximab for complete remission [11].

In summary, we report an unusual cause of ulceration in a systemically unwell infant which should be considered in the differential particularly when it has formed at sites of minor trauma. To our knowledge, only one other case has been reported in the literature to date in association with pneumonia and required both IL and IV corticosteroid therapy as well as Dapsone to induce remission [12].
